# A Systematic Review of Knowledge, Attitudes, and Factors Influencing HPV Vaccine Acceptance Among Adolescents, Parents, Teachers, and Healthcare Professionals in the Middle East and North Africa (MENA) Region

**DOI:** 10.7759/cureus.60293

**Published:** 2024-05-14

**Authors:** Sophia C Vincent, Safia Al Yaquobi, Aysha Al Hashmi

**Affiliations:** 1 Nursing, Sultan Qaboos University, Muscat, OMN; 2 Nursing, Sultan Qaboos University Hospital, Muscat, OMN; 3 Nursing, Oman College of Health Sciences – North Sharqiya, Sharqiya, OMN

**Keywords:** mena region, adolecents knowledge & attitude, parental knowledge and attitude, hpv vaccine hesitancy, hpv infection

## Abstract

Human Papillomavirus (HPV) infection is the most common sexually transmitted infection, affecting both men and women globally. Men and women are at risk of type HPV16 and HPV18 viruses leading to cervical, anal, vulvar, and oropharyngeal cancers. The HPV vaccines are highly effective in preventing various strains of HPV infection, and effective vaccines are available only in the Middle East and North Africa (MENA) region. Hence, this systematic review explored knowledge and attitudes toward HPV infection and HPV vaccination and factors influencing HPV vaccination uptake among the MENA populations.

Various databases, such as Medline, Science Direct, CINHAL, EBSCO, PsycINFO, and PubMed, were systematically searched to include English studies assessing knowledge, attitudes toward HPV infection, and factors influencing HPV vaccination acceptance in the MENA region. Twenty-two papers met the inclusion criteria. The number of participants ranged from 99 to 7223. While knowledge, attitude, and vaccine hesitancy association factors were generally reported in cross-sectional studies, the HPV vaccine acceptancy over time from longitudinal studies was mixed and inconclusive due to inadequate information on HPV infection and vaccination, the cost of the vaccine, cultural beliefs, and safety concerns. Results demonstrated that low HPV vaccine acceptance is due to a lack of HPV understanding among the MENA population, coupled with access issues. Factors such as awareness, confidence in vaccination, and involvement in decision-making positively influence vaccine uptake. Therefore, tailored programs addressing vaccine hesitancy in the MENA communities are necessary.

## Introduction and background

Human Papillomavirus (HPV) infection is the most common sexually transmitted infection worldwide, affecting about 80% of sexually active people. It is linked to various cancer types, with cervical cancer being the most notable. There are over 200 types of HPV, with about 40 transmitted through sexual activity. The most prevalent strains are HPV 16 and 18, which are responsible for almost 70% of HPV-related cancers [1]. Cervical cancer is the fourth most common cancer among women globally, with over 600,000 new cases and 300,000 deaths in 2022. Regions such as Southeast Asia, Central America, and sub-Saharan Africa have the highest incidence and mortality rates. As compared to developed countries, reports on HPV infection and human cancers are limited in developing countries, including the Middle East and North Africa (MENA) region.

Different strategies, such as screening and vaccination, are currently available to prevent cervical cancer. These strategies were found to be cost-effective in reducing morbidity and mortality related to cervical cancer. Currently, the 9-valent HPV vaccine (Gardasil 9v HPV), quadrivalent (Gardasil 4v HPV), and bivalent (2v HPV) are approved worldwide by the FDA, including the MENA region [2]. Since its availability in 2006, only a few countries have integrated the HPV vaccine into their national immunization schedules. In the MENA region, HPV vaccination is still emerging. Libya and the UAE are the only two countries with the HPV vaccine included in their health programs, and Turkey stands out as the only country with an organized screening program since 2013 [3]. On the other hand, countries such as Qatar, Algeria, and Morocco initiated HPV vaccination programs in their health settings but made them available to the population on demand [4].

However, most MENA countries do not screen for HPV infections due to stigmatization based on religious and traditional values, plausibly leading to small sample sizes and low incidence rates of HPV infection, thus miscalculating the actual number of cases. These disparities in immunization, screening, and treatment programs contribute to regional variations in cervical cancer rates [5]. None of the countries in the MENA region recommend the HPV vaccine to boys, as male circumcision is mandatory in these regions [6]. 

Understanding HPV infection and the HPV vaccine plays a crucial role in determining vaccine dissemination decisions. However, research on HPV vaccine uptake among the MENA population has been sparse since the vaccine's licensure in 2006. This scarcity is attributed to various factors, including language barriers, cultural disparities, legal concerns, religious beliefs, educational disparities, the limited availability of specialized health services, and a lack of awareness regarding HPV. Hence, this systematic review aims to fill this research gap by systematically analyzing published data on adolescents, parents, healthcare professionals, and teachers' knowledge, attitudes, and factors influencing the acceptance of the HPV vaccine to inform future initiatives and enhance HPV vaccine coverage.

Material and methods 

Data Search Strategy

**Figure 1:**
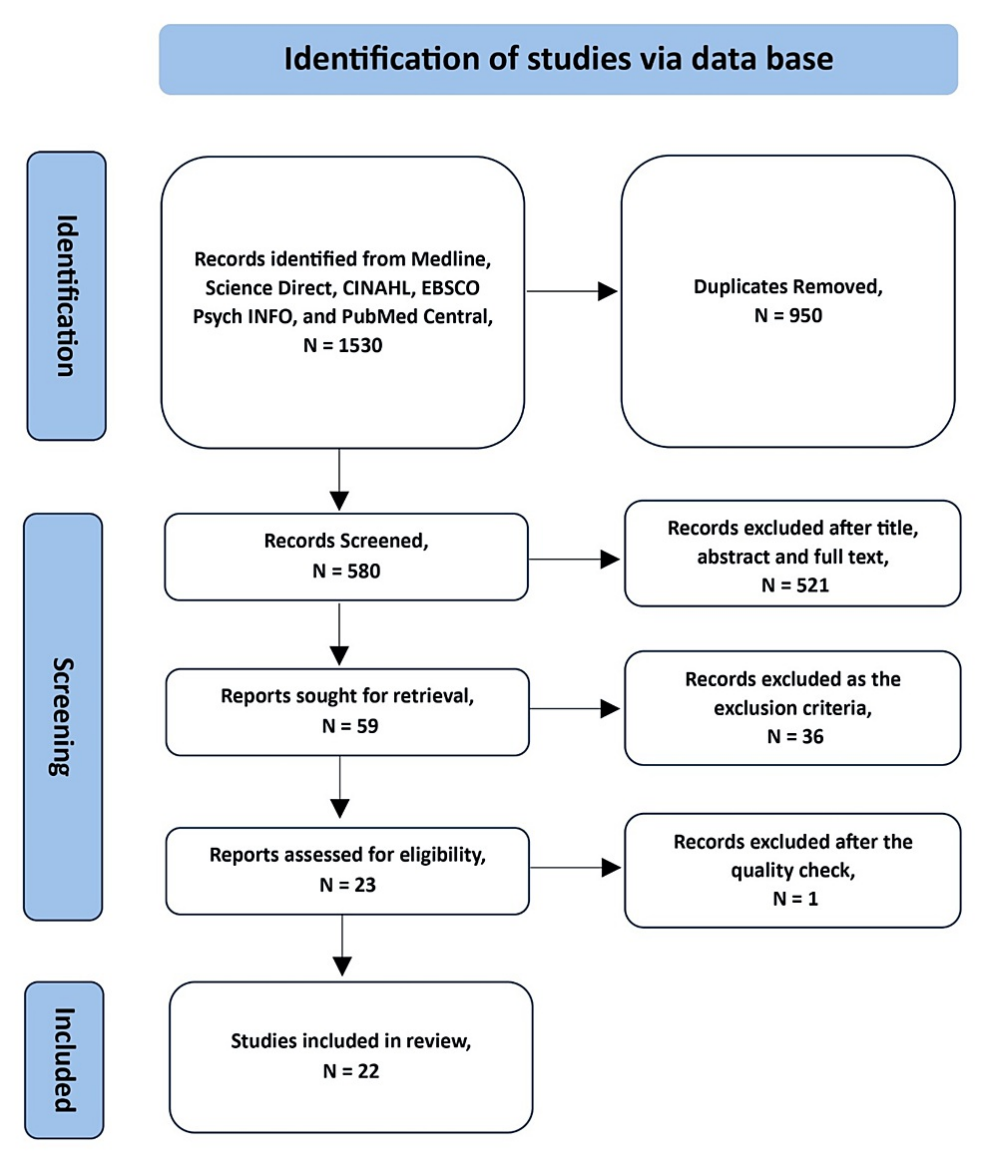
PRISMA flow diagram showing the study search and selection process PRISMA: Preferred Reporting Items for Systematic Reviews and Meta-Analyses

We conducted a systematic review of the peer-reviewed published literature using methods following the Preferred Reporting Items for Systematic Reviews and Meta-Analyses (PRISMA) guidelines (Figure 1) [7]. Two authors completed a thorough and rigorous selection procedure that comprised the analysis of article titles, abstracts, and full text. Medical subject headings (MeSH) terms (see Appendix A) were used to search for relevant articles in six bibliographic databases, including Medline, Science Direct, CINAHL, EBSCO, PsycINFO, and Pubmed Central. Some of the keywords searched alone or in combination were 'young adult,' 'parent,' 'HPV knowledge,' 'HPV vaccine hesitancy,' 'Cervical cancer,' and 'human papillomavirus.' An additional manual search was performed of the bibliographies of relevant studies identified from the database search. The team reviewed the articles found in the search and removed duplicates. 

Inclusion and Exclusion Criteria

The inclusion criteria encompassed studies reporting knowledge and perceptions of HPV infection and the HPV vaccine among the population of the MENA region, including both the general public and healthcare providers. The search aimed to include studies published between 2016 and 2023 in the English language without publication status restrictions. The exclusion criteria included case reports, reviews, editorials, letters to editors, commentaries, and studies on other vaccine hesitancies.

Data Extraction

We retrieved the full text of eligible studies for review and abstraction. We then created a detailed codebook for data collection. Data extraction tables for the article and quality assessment were developed and maintained in an Excel database (Microsoft Corp., Redmond, WA, USA). They were modified following discussions between three reviewers before data extraction. Data extracted included study location, study design, target population, sample description, and setting. Data extraction and validity assessment were conducted by two reviewers, with a third reviewer resolving the disputes. 

Appraisal for Methodological Quality

Two reviewers evaluated the strength and validity of the selected studies. A third reviewer was involved in resolving these differences. The quality of selected articles was assessed using the Strengthening the Reporting of Observational Studies in Epidemiology (STROBE) technique [8]. The STROBE score for the analyzed studies ranged from 18 to 26 points out of 29. The majority of the papers included in this review also failed to explain how missing data were addressed. The majority of the research studies also admitted that there were some flaws with the STROBE checklist.

## Review

Results 

The search identified 580 studies after removing duplicates. As a result of the title and abstract screen, 521 studies were excluded as they did not focus on knowledge and attitude towards HPV infection and HPV vaccine hesitancy. The full text of the remaining 59 articles was reviewed, leading to the exclusion of an additional 36 articles as the studies are not from the MENA region. This resulted in 23 articles being included in the review for data extraction (Figure 1), of which one study was excluded after quality analysis. Thus, a total of 22 studies were included in the review. Table 1 shows the main characteristics of the included studies, published between 2016 and 2023.

**Table 1:** Clinical characteristics and outcomes of the included studies HPV: Human papillomavirus, CC: Cervical cancer, STROBE:  Strengthening the Reporting of Observational Studies in Epidemiology

#	Author (year)	Study design	Country	Target population	Population size & education background	Tools	Results	Identified determinant factors associated with HPV vaccination	STROBE score		
1	Bencherit et al., (2022) [9]	Cross-sectional study	Algeria	20 to 29 years; male and female	715 males and females. Natural and life sciences, humanities, economics, sciences & technology, medical sciences	Self-administered questionnaire	Around 84% were aware of cervical cancer; 77.8% of medical students and 58.8% of natural and life science students had previous knowledge of HPV. The lowest level of knowledge was among the economics, humanities, and technology students. Around 13% were unaware of the causes for HPV infection; 26.6% were aware of early screening; 1.25% underwent Pap smear tests; and 58.4% of students reported never having heard of the HPV vaccine. Only 5.8% of students received the vaccine; 26.7% were willing to receive it; 21.5% were willing to pay for it; and 37.6% were unwilling to obtain it.	Not considering self at risk of HPV infection. Complacency, refusal of any vaccine. Not considering HPV as a common infection in Algeria.	20		
2	Yacouti et al., (2020) [10]	Cross-sectional study	Morocco	17 to 26 years	1087 General degree	Structured interviewer	Around 82% were aware of cervical cancer; 85.3% were aware or unaware of HPV; and 74% knew that it could be screened at an early stage. Around 82.8% and 92.2% were unaware of the Pap smear test and the HPV vaccine, respectively. About 67% were willing to receive the vaccine.	High cost of the vaccine, lack of perceived risk for cervical cancer, concerns regarding the safety and efficacy of the vaccine	22		
3	Sallam et al., (2021) [11]	Online survey	Jordan	>18 years; female	836 medical pharmacy and dental nursing students	Self-administered questionnaire	About 75% of medical students, 63.84% of dental students, 57.50% of pharmacy students, and 33.64% of nursing students had knowledge about HPV. Overall, 3.63% were vaccinated, 75.00% intended to be vaccinated, and only 16.03% were ready to pay for the vaccine.	Not considering themself at risk of HPV infection; not considering HPV as a common infection; inconvenient to be vaccinated	22		
4	Alsanafi et al., (2022). [12]	Cross-sectional survey	Kuwait	18 to 24 years	611 female students belonging to allied health, social & life sciences, public health, engineering & architecture, and humanitarian courses	Vaccine conspiracy belief scale (VCBS)	Around 50.9% to 84.0% had knowledge of HPV depending on their profession; 78.7% knew about the vaccination to prevent HPV infection; and 8.9% indicated the previous uptake of HPV vaccination. Higher vaccine knowledge was found among those older than 22 years (11.5%) and 4.2% among students younger than 22 years. Only 23.1% were willing to pay for the vaccine.	Concerns regarding safety and efficacy of vaccine; not considering themselves at risk; thought it inconvenient to get the vaccine; found visiting doctors uncomfortable; believed that HPV is not a common infection in the region	26		
5	Alsous et al., (2021) [13]	Cross-sectional survey	Jordan	≥ 18 years	504 university students	Questionnaire	About 40.5% were aware of the HPV vaccine's availability, 71.4% were aware it should be given between 11 and 29 years of age, 65.9% found it acceptable to receive the HPV vaccine, and 54.0% knew it could be given to boys. Around 21.0% knew about screening for HPV; less than one-third of students (30.8%) were aware that the vaccine cannot be given to a woman already having an HPV infection; and 19.0% knew that three doses are required for protection in women.	Inadequate information about CC and the HPV vaccine; no emphasis in the curriculum; lack of educational campaigns	22		
6	Aldawood et al.,(2022) [14]	Cross-sectional study	21 to 23 years	Saudi Arabia	403 King Saud University College of Applied Medical Sciences	Questionnaire	The highest proportion of knowledge is in the College of Medicine (81.2%), while the lowest number of students from the College of Applied Medical Sciences (24.1%); HPV vaccine hesitancy is 70.6% from the College of Applied Medical Sciences with the highest proportion being among males.	Being sexually inactive (39.4%) and lacking sufficient knowledge about vaccines (39.7%). Males (47.1%) believed they did not need the vaccine compared to females (22.4%).	20		
7	Farsi et al., (2018) [15]	Cross-sectional study	Saudi Arabia	17 to 25 yrs	517 male medical students	Self-administered questionnaires	Around 58.4% never heard of the HPV vaccine; 48.9% were interested in receiving the HPV vaccine; 8.7% reported already having received it; 55.2% planned to discuss it with their female patients; 56.9% planned to discuss it with their male patients	Perception of not needing the HPV vaccine, not being sexually inactive, and the lack of HPV vaccine knowledge.	22		
8	Zakhour, (2017) [16]	Cross-sectional survey	Lebanon	Parents of children aged 3 to 18 years	306 Lebanese parents of public & private school children	Self-administered questionnaires	Parents of both public & private schools were in favor of the vaccine: 60% were not planning to give the HPV vaccine to their children;95% of participants stated getting information on vaccines from doctors; 13.1% thought that vaccines are not safe. An effect of gender on vaccine acceptance was noted among mothers vs. fathers and daughters vs. sons.	Lack of recommendation by pediatricians. Lack of knowledge about the vaccine.	24		
9	Hendaus et al.,(2019) [17]	Cross-sectional study	Qatar	Parents of children 9 to 18 years	334 parents aged 20 to 39 years	Self-administered questionnaires	Around 60% of the parents were not aware that HPV can cause cervical and genital cancer; 4% of parents stated that their children's primary care physicians never mentioned that such a vaccine exists; 20% of the participants were not convinced about the HPV vaccine; 13% stated that the vaccine has been developed for marketing goals; 15% preferred not to talk to children about a vaccine related to sexual relations and the rest mentioned that cancer is a disease for old people.	Safety of the vaccine and vaccine efficacy; cultural beliefs; received no recommendation from their physicians	20		
10	Saqer et al.,(2017) [18]	Observational cross-sectional study	Sharjah	36 to 55 yrs	400 parents	Self-administered questionnaire	About 78.3% of the population had heard of CC; 41.3 % of HPV; and 36.5% of the HPV vaccine. Around 92.9%, of parents were willing if the Ministry of Health (MOH) recommended the vaccine.	Vaccine efficacy, vaccine not included in the immunization schedule or not recommended by MOH	22		
11	Al Alawi et al., (2023) [19]	Cross-sectional quantitative study	Oman	Parents & healthcare providers	13,21,952 parents and 369 healthcare providers	Self-administered questionnaire	Around 49% of healthcare workers were aware of HPV infection; 51% of parents were aware of HPV infection; and only 37.4% believed the vaccine was safe.	Lack of sexual and reproductive health education in Omani schools; side effects rather than efficacy; cost of the vaccination; absence of HPV vaccination in Oman’s national immunization schedule; cervical screening at the primary healthcare level	22		
12	Anfinan et al., (2018) [20]	Cross-sectional study	Saudi Arabia	Not specified	2000 physicians	Questionnaire	Around 62.0% had adequate knowledge; 7.6% were immunized; 41.2% accepted to receive the vaccine; and 77.6% felt favorable in vaccinating their children. Overall negative attitude regarding vaccines was typically associated with male, older, senior consultants. The Ob/Gyn specialty physicians were aware of HPV infection and had a positive attitude towards HPV vaccination.	Lack of knowledge about the vaccine being sexually inactive till marriage	21		
13	Qaqish et al., (2023) [21]	Cross-sectional study	Jordan	Physicians from Jordanian health sectors	412 physicians	Questionnaire	Around 97.8% of them had heard of HPV, it was higher among female physicians, and the youngest physicians. Physicians' knowledge of different non-sexual routes of HPV infection was extremely low.	No extramarital activity; maintaining 'virginity'; cost of the vaccine	20		
14	Rezqalla et al., (2020) [22]	Cross-sectional study	Kuwait	20 to 61 years	1341 school teachers	Questionnaire	The prevalence of unawareness of HPV vaccine availability was 88%. Around 83.8.% were unvaccinated against HPV infection; 18.6% of the participants were unaware of cervical cancer; 9.4% had a personal and/or family history of cervical cancer; and 47.1% were unaware of Pap smear tests. Female schoolteachers employed in public schools were significantly more likely to be unaware of HPV vaccine availability compared to those working for private schools.	Limited access to reproductive health information; cultural beliefs; belief that vaccination is not necessary, vaccines are unsafe, and/or being against all vaccinations	22		
15	Husain et al., (2019) [23]	Cross-sectional study	Bahrain	Males and females aged 18 to 65 years	268 women and 140 men	Self-administered questionnaire	Around 13.5% of males and 50% of females had knowledge & awareness of HPV infection and vaccination; 69% were aware that the virus can lead to cervical cancer and unsafe sexual practices can increase the probability of getting HPV infection; 60%thought that the vaccine is unsafe.	Lack of public education regarding the virus and the absence of HPV vaccination in the national immunization schedule. Side effects concerns. Trust issues between couples.	22		
16	Harper et al.,(2019) [24]	Community-based surveys	MENA region	18 to 34 years	507 adult men	Online questionnaire	Around 45% had no knowledge of the HPV vaccine and 80% of men were not vaccinated. The MENA men who are most likely to vaccinate are younger, US-born, a student, and have a college education. Prior work highlights similar cultural influences on health behaviors among Arab Americans. Single marital status was only associated with HPV vaccination initiation.	Community input, support, and implementation to increase this community's male HPV vaccine uptake	23		
17	Al Shdefat et al.,(2022) [25]	Cross-sectional survey	UAE	20 to 65 years	400 Emirati men	Self-administered questionnaire	Emirati males had a 37% acceptance rate; 20.5% believe it’s safe, while 22.6% of respondents did not recommend it as they thought it is not safe; 41% of the respondents will not recommend it even if it was recommended by the religious authority while 2.1% were ready to recommend if backed by religious authority.	Around 23.6% of respondents felt it was culturally unacceptable and 11.3%of respondents said that vaccines are culturally undesirable, while 2.1% stated that vaccines are religiously inappropriate, and 5.4% stated that women are often the least worried about their health.	25		
18	Alsous et al., (2021) [26]	Cross-sectional survey	Jordan, Qatar, the United Arab Emirates (UAE) and Iraq	≥18years	2804 1216-Jordan, 397 - Qatar, 606 - UAE, 585 - Iraq	Self-administered questionnaire	Around 26.9% knew that the HPV infection is a sexually transmitted disease; 25.6%) were aware that the vaccine does not protect against all types of CC; 21.3% of participants were aware that HPV causes some side effects such as headache and nausea; and 20.3% knew that the HPV vaccine can prevent CC and decrease the chance of having changes in the Pap smear test. Moreover, the proportion of participants who were aware that the target population for vaccination was both males and females and that taking the vaccine would not infect the recipient was 20.7% and 23.6%, respectively.	Poor knowledge; inadequate information about CC and HPV vaccine. The other main concern was about the side effects, efficacy, and cost of the vaccine.	24		
19	Elshami et al., (2022) [27]	Cross-sectional study	Palestinian	15 to 49 years	7223 women	Self-administered questionnaire	Only 0.5% demonstrated good awareness; 8.1% had heard about HPV; 89.1% would agree to receive the HPV vaccine if it was given for free; 91.3% would agree for their daughters to receive the HPV vaccine; and 40 87.0% also if it incurred a cost.	Lack of the HPV vaccination in the national immunization program. Absence of public education interventions.	23		
20	Namoos et al., (2021) [28]	Cross-sectional quantitative study	Egypt	18 to 55 years	99 married women participants,	Personal interviews	Around 80% of women had a significant lack of awareness regarding HPV infection and the vaccine, particularly among less educated and highly religious communities.	Education and religiosity were identified as important predictors of HPV awareness, with higher education levels associated with a greater likelihood of being informed about HPV.	24		
21	Darraj et al., (2022) [29]	Cross-sectional study	Saudi Arabia	18 and older	569 females	Semi-structured validated questionnaire	About half of the participants denied that HPV is a common sexually transmitted infection. Further, 53% were interested in the HPV vaccine, and 63% of participants acknowledged that the HPV vaccine could prevent warts and cervical cancer.	Around 30% of the participants opposed the vaccine due to religious reasons.	22		
22	Turki et al., (20023) [30]	Cross-sectional study	Maakha, Saudi Arabia	Females >16 years attending the PHC	534 female	Questionnaire	Around 1.6% to 59.6% highlighted the need for educational programs 48.4%. A majority of participants expressed willingness to receive the HPV vaccine if offered by the healthcare sector at no cost.	Concerns included fear of injection (27.7%), cost (23.2%), and potential refusal from family or community (9.7%). A significant proportion believed that there is a need for educational sessions to increase awareness about the HPV vaccine in their community (82.8%) and that increased knowledge about HPV vaccines would lead to greater acceptability (83.9%).	23		

Of the 22 cross-sectional articles, five were conducted in Saudi Arabia. Other studies were conducted in Jordan (n = 3), Kuwait, the UAE, the MENA region (n = 2), Algeria, Lebanon, Qatar, Oman, Bahrain, Palestine, Egypt, and Morocco (n = 1). The total number of participants in the included studies ranged from 99 to 7223, encompassing both genders and some studies not specifying gender. The ages of the interviewees ranged from 17 to 65 years. Studies were conducted among adolescents, parents, MENA region men, doctors, and teachers. Intervention settings included medical colleges, universities, non-professional courses, and primary health centers.

The study instruments included the Parent Attitudes about Childhood Vaccines (PACV) questionnaire and the Vaccine Conspiracy Belief Scale (VCBS). The remaining studies used self-administered or previously used questionnaires prepared by the researchers. Knowledge, attitude, and HPV vaccine hesitancy in the MENA region were discussed in four thematic categories.

Knowledge and Attitude Regarding HPV Infection and HPV Vaccination Acceptance Among Adolescents

The study discovered that teenagers had varying levels of understanding and attitudes concerning cervical cancer and HPV vaccination. A sizable proportion had prior awareness of cervical cancer (60% to 84.6%) and HPV infection (64.7%). Those with backgrounds in medical and life sciences, married people, those over the age of 30, women, and those with master's or post-graduate degrees demonstrated greater levels of expertise. Interestingly, while a large number (84.6%) were aware of cervical cancer, there was a huge knowledge gap, with 85.3% ignorant of the link to HPV. Awareness of screening measures such as the Pap smear test (82.8%) and the HPV vaccination (92.2%) was equally poor. However, opinions about HPV vaccination were usually low across all teens. Attitudes towards HPV vaccination were generally low in all adolescents, with only 3.5% to 5.8% having received the vaccine and 26.7% to 50% expressing willingness to receive it. Very few, only 21.5%, were willing to pay for the vaccine, while 37.6% to 70% expressed unwillingness to obtain it [9-12].

Despite efforts to increase awareness, there's still a significant knowledge gap concerning HPV vaccines among adolescents, particularly regarding their availability and importance. Vaccine hesitancy, especially among certain groups like males and students in the College of Applied Medical Sciences, is evident [11]. Research suggests that adolescents, particularly males, often underestimate their susceptibility to HPV due to sexual inactivity and perceive it as primarily a concern for females [13]. Lack of awareness about HPV as a common infection, considering themselves and their region as not at risk, vaccine cost, safety, complacency, and efficacy contribute to hesitancy. Some adolescents also felt uncomfortable visiting doctors. Furthermore, there's insufficient focus on cervical cancer and the HPV vaccine in educational campaigns and a lack of integration of this topic into the academic curriculum [14,15].

Knowledge and Attitude Regarding HPV Vaccine Acceptance Among Parents in the MENA Region

Research conducted in four countries in the MENA region revealed that a significant portion (60% to 78%) of parents whose children attend private and public schools are aware of cervical cancer. Despite receiving information about the HPV vaccine from healthcare providers, a staggering 95% of parents either had no knowledge of its causes or had never even heard of the HPV screening test [16]. Interestingly, only a small minority of parents, around 13%, suspected that the HPV vaccination was developed for commercial gain [17].

An overwhelming 92.9% of parents expressed willingness to vaccinate their children if mandated by the Ministry of Health [18]. The identified factors influencing the parent's knowledge and attitude were not wanting to discuss sexual health with their children and cultural beliefs [19]. Additionally, fear about vaccine safety, a lack of awareness about HPV and its linked cancers due to limited public campaigning, a lack of doctor recommendations, and not including the HPV vaccine in the national immunization was the observed parent concerns in all the studies.

Knowledge and Attitude Regarding HPV Vaccine Acceptance Among Health Care Professionals and Teachers in the MENA Region

Research has been carried out on how healthcare providers in the MENA region perceive and handle cervical cancer by understanding their knowledge and attitudes regarding HPV infection and vaccinations. These studies found significant gaps in knowledge among physicians, influenced by factors such as their work setting, specialization, age, and gender. Particularly, primary care physicians (62%) showed limited awareness of HPV infection, vaccine efficacy, dosing schedules, and target age groups for vaccination, with a majority reluctant to vaccinate their daughters. Conversely, 97.8% of female physicians, including gynecologists and pediatricians, were overwhelmingly supportive of vaccination and actively provided information to the public [18]. Conversely, older male physicians, especially senior consultants, exhibited negative attitudes toward HPV vaccination and demonstrated limited knowledge [20-21]. All the studies highlighted various influencing factors, such as time constraints, parental beliefs, controversy surrounding discussions on sexual health, financial limitations, and personal beliefs.

Only one study elucidates a critical gap in understanding and awareness regarding HPV infection and vaccination uptake among school teachers in the MENA area. The findings underscore the importance of comprehensive education and awareness campaigns aimed at educators, who can serve as vital conduits of information to students and the broader community. Only 18.6% of teachers were aware of the HPV infection. Half of the teachers were unaware of the Pap smear exam. Factors such as a lack of complete information, cultural and religious views, and stigma connected with sexually transmitted illnesses may all contribute to this restricted understanding [22].

Knowledge and Attitude Regarding HPV Vaccine Acceptance Among Men and the General Public

Eight research studies were conducted to understand and address the general public, of which two were exclusively conducted among MENA men to understand HPV awareness and attitudes towards the HPV vaccine. Unfortunately, only 13.5% to 35% of men were aware of HPV infection, and 20% of men were aware that HPV is a sexually transmitted disease and can lead to cervical cancer, oral cancer, rectal cancer, and oropharyngeal cancer. More than half of the men mentioned that the HPV vaccine is unsafe [22-24]. Also, the vaccine acceptance rate was found to be only 37%. Interestingly, Arab American men residing in the MENA region who were born and raised in the US tend to be the primary recipients of HPV vaccination. However, single and unmarried men often perceive vaccination as unnecessary. Men's reluctance to receive the HPV vaccine, despite recommendations from the Ministry of Health or religious leaders, highlighted the complex interplay of cultural, social, and personal beliefs in healthcare decision-making [25].

The cross-sectional study conducted across five MENA regions (Jordan, Qatar, the UAE, and Iran) revealed concerning gaps in awareness regarding HPV and the HPV vaccine, revealing that only 26.9% of respondents recognized HPV as a sexually transmitted disease. Around 25.6% understood that the HPV vaccine does not protect against all types of cervical cancer. Just 20.3% knew that the HPV vaccine could prevent cervical cancer and reduce abnormal Pap smear tests. The same result was observed in the percentages recognizing that both males and females should be vaccinated [26]. The HPV survey conducted in Palestine revealed mixed awareness among females. While 8.1% demonstrated good awareness of HPV infection, only 16.6% knew it as a sexually transmitted disease, and 9.3% recognized the possibility of transmission from mother to newborn. However, a majority (62.1%) correctly identified that HPV can infect both males and females. Awareness of the HPV vaccine was low, with only 8.1% having heard of it. Nevertheless, an encouraging 89.1% of women expressed willingness to receive the vaccine if provided free of charge [27].

Surprisingly, women with a lack of HPV infection knowledge showed a positive attitude about the HPV vaccine, particularly within less educated and highly religious communities. About 53% of women expressed interest in HPV screening and HPV vaccination, and 63% of participants acknowledged the vaccine's potential to prevent warts and cervical cancer. The identified factors for the dearth of awareness were the lack of educational programs in the region, religious belief, vaccine cost, fear of side effects, and fear of pain as it is not included in the national immunization schedule [28-29].

Discussion

We conducted a systematic review to assess knowledge, attitude, and factors influencing HPV vaccine hesitancy among the MENA population. Our goal was to better describe common target populations, as previous systematic reviews have identified either HPV knowledge, HPV vaccine hesitancy, or determinants for vaccination among the general population [30-31]. Hence, we focused on investigating the different studies conducted among adolescents and the elderly population to narrow the scope of the study and identify gaps in existing knowledge. 

Furthermore, this comprehensive viewpoint enables a deeper examination, taking into account developmental aspects, distinctions between generations, and possible variations in outcomes related to age. Ultimately, this methodical review of existing literature forms the basis for crafting a targeted and knowledgeable study that adds significant value to the field. Although HPV vaccines have been proven safe and effective in preventing HPV-associated infections and cancers when given early in adolescence, vaccination uptake rates remain low globally, irrespective of demographic variables such as age, education, profession, and gender [32]. And the same result was observed among the MENA population. 

Adolescents in the MENA region often believe they are not susceptible to HPV infection due to cultural and moral values. However, this perception overlooks the reality of HPV transmission beyond sexual activity. Cultural taboos, misinformation, and concerns about vaccine efficacy contribute to a significant knowledge gap. Addressing this gap requires targeted education campaigns to dispel myths and improve access to accurate information and affordable vaccines. Similar findings were reported by Luo et al. in China, Radomski et al. in North Macedonia, and Ogboul in Nigeria [33-35]. 

Similarly, men from the MENA region showed reluctance towards receiving the HPV vaccine. This hesitance may be influenced by several factors, including the demographics of the sample population, such as their age and sexual activity levels. Older individuals with more sexual experience might have a heightened understanding of HPV and its risks, which could increase the likelihood of them accepting the vaccine. This observation aligns with findings from a study involving Latino Americans, where researchers found that awareness about HPV and its vaccination tended to increase with age and sexual experience [36]. The insufficient knowledge of HPV in men may be due to campaigns targeting females only, with a clear focus on cervical cancer. Thus, knowledge is not always predictive of vaccine acceptance or uptake [37]. In contrast, Songthap et al. discovered that men in Thailand have a high level of knowledge about HPV infection and HPV vaccination [38]. 

It's also crucial to understand parental perspectives on the HPV vaccine to effectively encourage vaccination uptake in their children. Parents wield significant influence in healthcare decisions, so their knowledge, attitudes, and acceptance are pivotal. The success of any immunization program hinges on parental understanding of the illness and their attitudes toward vaccination [39]. Unfortunately, in the MENA area, parental unawareness is a huge impediment. Numerous studies have repeatedly shown that many parents in this area are unaware of HPV and its link to cervical cancer, resulting in poor vaccination rates. Our analysis also shows that parents are hesitant to vaccinate their children against HPV due to religious and cultural constraints, a lack of understanding of HPV and its associated risks, concerns about the vaccine's safety and effectiveness, and, most importantly, limited access to healthcare facilities. Furthermore, our findings highlight the crucial role of healthcare practitioners and community leaders in affecting parental decisions about HPV vaccination. These findings are consistent with cross-sectional research done in China by Youqin et al., which likewise highlighted a comparable lack of understanding among parents of teenage daughters regarding HPV and its vaccine [40]. Similarly, research in Serbia revealed that while 76.6% of mothers were open to vaccinating their daughters, concerns about side effects and a lack of knowledge remained significant barriers [41]. Thus, community-based interventions are crucial for addressing vaccine safety concerns and dispelling prevalent misinformation among parents in the MENA region.

The knowledge of physicians about the HPV vaccine in the review was observed to be very low, especially among general physicians, dermatologists, and venereologists. In addition, it was found that the state of knowledge depended on the age, the place of employment (a higher level of expertise among university employees), and the number of daily patients (higher among doctors seeing 20 to 29 patients a day). Also, healthcare professionals were reluctant to address public concerns regarding HPV infection or HPV vaccination due to the cultural norms surrounding sexual activity before marriage. Similarly, a study by Rosenthal et al. highlights some significant factors such as time constraints, administrative burdens, concerns regarding vaccine effectiveness and safety, reluctance from patients to adhere to follow-up procedures after vaccination, and even societal misconceptions such as the belief that vaccination might promote risky sexual behavior [42].

Teachers can play a crucial role in successful vaccination programs, especially in the absence of a school health nurse. Our review observed that teachers had moderate knowledge about HPV infection and HPV vaccination. However, it's understandable that many teachers might not have extensive knowledge, as it's not typically part of their formal training or job responsibilities. Hence, it could be beneficial to provide teachers with resources and training on HPV and vaccination so they can better support their students and families in making informed decisions about their health. This could ultimately contribute to higher vaccination rates and better public health outcomes [43-44].

The implications of these findings are significant, as parents, healthcare professionals, and educators play a crucial role in shaping societal attitudes and behaviors toward vaccination. Therefore, there is a critical need to enhance education on HPV infection and vaccination among the MENA population. This can be achieved through targeted training programs, workshops, and educational campaigns aimed at increasing awareness, addressing misconceptions, and promoting the importance of vaccination in preventing HPV-related disease. 

## Conclusions

The present systematic review explores the main factors influencing the MENA population's decision to vaccinate against HPV observed in 22 studies. Results indicate that knowledge about HPV infection could positively influence vaccination rates. There is a need to address the knowledge gaps by organizing HPV vaccination campaigns and sexual education programs for the MENA population, regardless of the vaccination uptake intent. Other aspects affecting vaccination uptake were fear of side effects, insufficient information, and the belief that the chances of being infected are low. Therefore, we recommend future research focusing on strategies to improve knowledge and awareness impacting vaccination decisions and behavioral change to expand the uptake of vaccines among the MENA population. This present comprehensive analysis can be the foundation for exploring diverse programs to mitigate vaccination addressability.

## Appendices

Appendix A

Detailed Search Strategy

A search was conducted on the databases CINHAL, PubMed, Medline, Google Scholar, PsycINFO, and Science Direct for articles published between 2016 and 2023. 

MeSH terms used for the search: (“Cervical Neoplasms” [Mesh] OR "Papillomavirus Infections"[Mesh] OR HPV [tiab] OR “cervical cancer” [tiab] OR “papillomavirus” [tiab] OR cervix[tiab] OR “cervical neoplasm*” [tiab] OR “cervical tumo*” [tiab] OR “cervical malignan*” [tiab]) AND (“Mass vaccination”[Mesh] OR "Vaccination"[Mesh] OR "Neoplasms/prevention and control"[Mesh] OR vaccin*[tiab] OR prevent* [tiab] OR immuni* [tiab] OR “Mass screening”[Mesh] OR "Early Diagnosis"[Mesh] OR "Early Detection of Cancer"[Mesh] OR screen*[tiab] OR “knowledge”[tiab] OR “attitude ”[tiab] OR vaccine hesitancy " parents/adolescents"[Mesh]OR "MENA population.
